# Role of Selected miRNAs as Diagnostic and Prognostic Biomarkers in Cardiovascular Diseases, Including Coronary Artery Disease, Myocardial Infarction and Atherosclerosis

**DOI:** 10.3390/jcdd8020022

**Published:** 2021-02-19

**Authors:** Rashid Mir, Imadeldin Elfaki, Naina Khullar, Ajaz Ahmad Waza, Chandan Jha, Mohammad Muzaffar Mir, Shamsu Nisa, Babar Mohammad, Tahir Ahmad Mir, Mohsin Maqbool, Jameel Barnawi, Salem Owaid Albalawi, Faisel M. Abu-Duhier

**Affiliations:** 1 Prince Fahd Bin Sultan Research Chair, Department of Medical Lab Technology, Faculty of Applied Medical Sciences, University of Tabuk, Tabuk 71491, Saudi Arabia; jbarnawi@ut.edu.sa (J.B.); fabu-duhier@ut.edu.sa (F.M.A.-D.); 2 Department of Biochemistry, Faculty of Science, University of Tabuk, Tabuk 71491, Saudi Arabia; ielfaki@ut.edu.sa; 3 Department of Zoology, Mata Gujri College, Fatehgarh Sahib, Punjab 140406, India; naina306@gmail.com; 4 Department of Microbiology, Government Medical College (GMC), Srinagar, Kashmir, J & K, Srinagar 190010, India; ajazahmad09@gmail.com; 5 Department of Human Genetics, Punjabi University, Punjab, Chandigarh 147002, India; chandujha58@gmail.com; 6 Department of Basic Medical Sciences (Biochemistry) College of Medicine, University of Bisha, Bisha 61922, Saudi Arabia; mirmuzaffar11@gmail.com; 7 Department of Obstetrics & Gynaecology, Sher-i-Kashmir Institute of Medical Sciences, J & K, Srinagar, Soura 190011, India; drshamsu32@gmail.com; 8 Department of Radiodiagnosis and Imaging, Government Medical College (GMC), Srinagar, Kashmir, J & K, Srinagar 190010, India; babarmohmad000@gmail.com; 9 Inderprastha Dental College & Hospital, Ghaziabad, Uttar Pradesh, New Delhi 201010, India; tahirmir0814@gmail.com; 10 Dana Farber Cancer Institute, Boston, MA 02215, USA; mohsin13r@gmail.com; 11 Department of Cardiology, King Fahd Special Hospital, Tabuk 71491, Saudi Arabia; sal-wabsy@moh.gov.sa.com

**Keywords:** coronary artery disease, CAD, cardiovascular diseases, myocardial infarction, MI, acute myocardial infarction, AMI, microRNA, miRNA, miR

## Abstract

Cardiovascular diseases are the leading cause of death worldwide in different cohorts. It is well known that miRNAs have a crucial role in regulating the development of cardiovascular physiology, thus impacting the pathophysiology of heart diseases. MiRNAs also have been reported to be associated with cardiac reactions, leading to myocardial infarction (MCI) and ultimately heart failure (HF). To prevent these heart diseases, proper and timely diagnosis of cardiac dysfunction is pivotal. Though there are many symptoms associated with an irregular heart condition and though there are some biomarkers available that may indicate heart disease, authentic, specific and sensitive markers are the need of the hour. In recent times, miRNAs have proven to be promising candidates in this regard. They are potent biomarkers as they can be easily detected in body fluids (blood, urine, etc.) due to their remarkable stability and presence in apoptotic bodies and exosomes. Existing studies suggest the role of miRNAs as valuable biomarkers. A single biomarker may be insufficient to diagnose coronary artery disease (CAD) or acute myocardial infarction (AMI); thus, a combination of different miRNAs may prove fruitful. Therefore, this review aims to highlight the role of circulating miRNA as diagnostic and prognostic biomarkers in cardiovascular diseases such as coronary artery disease (CAD), myocardial infarction (MI) and atherosclerosis.

## 1. Introduction

According to World Health Organization http://www.who.int/cardiovascular_diseases (accessed on 2 October 2021), coronary artery disease (CAD) is the major cause of death in the world, amounting to about 31% of deaths worldwide. Thus, its earlier and authentic diagnosis can help prevent this disease and may aid in the treatment of patients. Many conventional diagnostic and prognostic biomarkers that can predict heart-related diseases are already available, which include the highly sensitive troponins, NT-pro BNP, left ventricular ejection fraction and the Systematic Coronary Risk Evaluation (SCORE) that provides a 10-year prediction of the risk associated with severe heart disease [1]. However, these methods serve well only in case of primary preventions and fail to give authentic results when applied to people who already have heart disease (preexisting heart patients). Thus, there is a need for better biomarkers that could work in case of secondary preventions. In the present context, an attempt has been made to evaluate various miRNAs as cardinal biomarkers for the diagnosis and prognosis of CAD [2]. MiRNA is a non-coding, small (up to 22 nucleotides long), single-stranded RNA molecule that is naturally equipped with properties of silencing RNA and regulating gene expression at the post transcriptional level. These miRNAs complementarily bind in the 3′ untranslated region (3′UTR) of the target mRNA and cause downregulation. It has been reported that microRs constitute up to 5% of human genome, and that about one third of the protein-coding genes is regulated by miRNAs [3].

**Figure 1:**
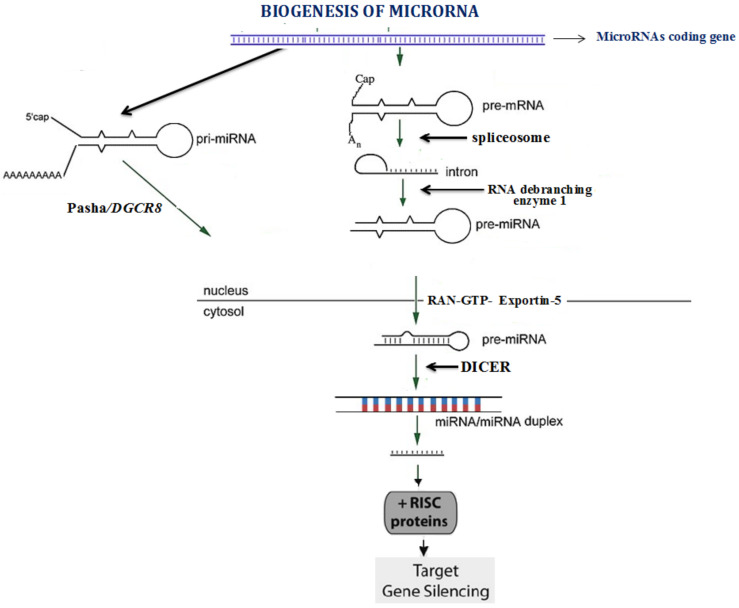
Biogenesis of microRNA.

In the canonical pathway of miRNA biogenesis, the miRNAs are transcribed from DNA into primary microRs (pri-miRNAs) and catalyzed by RNA polymerase II (pol II), as depicted in Figure 1. The RNA polymerase III (pol III) can also catalyze the transcription of the miRNAs that are involved in cell cycle and growth. The pri-miRNAs are processed into the precursor-miRNA (pre-miRNA) by the microprocessor complex [4]. The microprocessor complex is composed of the ribonuclease III enzyme, Drosha and an RNA-binding protein DiGeorge syndrome critical region 8 (DGCR8), as depicted in Figure 1. The pre-miRNA is translocated to the cytoplasm by the exportin 5 (XPO5)/RanGTP complex [5]. In the cytoplasm the terminal loop of the pre-miRNA is removed by the RNase III endonuclease, Dicer, resulting in the formation mature miRNA duplex. The miRNAs regulate the gene expression via binding to specific sequences within the 3′ UTR of the target mRNAs. This binding results in deadenylation and decapping of the target mRNA and consequently the translation is repressed. The miRNAs also have been reported to bind the 5′ UTR of the target mRNAs, which causes silencing of the gene expression or bind the promoter region, which enhances the transcription.

The miRNAs have been known to play a crucial role in pathological processes relating to coronary artery disease [5]. They are potent biomarkers as they can be easily detected in body fluids (blood, urine, etc.) due to their remarkable stability and are present in apoptotic bodies and exosomes [6]. These are released as paracrine factors by both living and dying cardiomyocytes and thus these may act as effective biomarkers in predicting heart attack. Several studies have investigated the association between miRNA gene variations and susceptibility to various diseases, such as diabetes, atherosclerosis and coronary artery disease [7,8]. Several recent studies reported association of the genetic variants with different metabolic diseases in different genes, including the seed region of the miRNAs [8,9,10,11,12,13,14]. Our earlier reports indicated the potential association of the miRNAs gene polymorphism with the risk of developing CAD, as well as its association with different cardiometabolic phenotypes to stratify the CAD burden in the general population [7,8,12,15,16]. The review aims to present an authentic update on the molecular mechanisms of miRNA’s role in the pathogenesis of CAD and its associated complications. It will also highlight the individual potential of different mRNAs as diagnostic and prognostic markers of these diseases.

## 2. Role of miRNAs 19b-3p in CAD

MiR-19b is located on chromosome 13 and is an important constituent of the miRNA-17-92 cluster, comprising of miR-17, 18a, 19b, 20a and 92a. Studies have shown a decrease in the amount of miR-19b in serum as well as myocardium of cardiac patients than in controls [17]. Several studies reported an association of miR-19b deregulation with increased myocardial enzyme lysyl oxidase (LOX) enzyme expression. Cardiac function is directly affected by extracellular matrix (ECM) composition, and alterations of the ECM contribute to the progression of heart failure. It is indicated that excess synthesis and activation of LOX in HF patients increase collagen cross-linking, significantly increasing collagen resistance to degradation by matrix metalloproteinases (MMPs), leading to increased stiffness in left ventricular chamber and thus leading to diastolic dysfunction. Thus, a reduced miR-19b expression leads to increased myocardial LOX protein and CCL and left ventricular stiffness in heart patients [18]. To date, only the ratio between C-terminal telopeptide of collagen type I to matrix metalloproteinase-1 is an approved biomarker for myocardial fibrosis, but now circulating miR-19b could serve as a cardinal biomarker. Many lines of evidence justify their involvement in ageing-related heart failures. Its secretion rapidly increases during apoptosis and hypertrophy of the cardiomyocytes involving atrogin-1 and MuRF-1 [19]. MiR-19b downregulates the expression of antihypertrophic factors Atrogin 1 and muscle ring finger protein 1. It also causes hypertrophy by inducing NFAT (calcineurin/nuclear factor of Activated T cells) and P13K. It is confirmed in various studies pertaining to atherosclerosis that miR-19b suppresses peroxisome proliferator-activated receptor γ coactivator 1α (PGC-1α), which leads to malfunctioning of endothelial cells [20]. It thus activates the accumulation of foam cells and macrophages; it further targets ATP-binding cassette transporter A1 (ABCA1) and causes development of aortic atherosclerosis. MiR-19b also targets pro-coagulant protein tissue factor (TF) as an antithrombotic agent, represses the α-crystallin-B encoding gene and weakens the Bim gene (pro-apototic gene) expression, and thus promotes cell survival [21]. Furthermore, the miR-17-92 cluster (that includes miR-19b) promotes survival, activation and proliferation of CD4^+^ T cells and hence regulates heart-related disorders. It induces inflammation mediated by Th17 and is also known to inhibit Th2. MiR-19b suppresses the expression of the TGFβRI and TGFβRII genes, thus downregulating the fibrogenesis process [22]. MiR-19b is regulated by the cMyc gene [23].

## 3. Role of miR-186-5p in CAD

MiR-186, miR-132 and miR-150 are differentially expressed in patients with unstable angina (UA), patients with chest pain of non-cardiac origin and healthy controls, respectively. Experimental evidence shows that the relative levels of miR-186-5p increased significantly in serum and conversely decreased in myocardial tissue. MiR-186-5p has been shown to induce atherogenic lipid formation. It also increased the proinflammatory cytokines production for progression of atherosclerotic lesions, plaque vulnerability and lesions [24]. MiR-186-5p may also be involved in hypoxic response and glucose metabolism, leading to acute coronary syndrome; however, experimental confirmations are still pending. MiR-186-5p, miR-134-5p and miR-19b-3p are all reliable biomarkers in the sense that their levels are not affected significantly by anticoagulant heparin and medicines for AMI (nitrates, beta blockers, aspirin, etc.) [25]. All three of these miRNAs get upregulated in the early phase of AMI.

Some studies have verified that miR 186-5p shows its peak expression usually 4 h after AMI. Thus, these three miRNAs together compositely give more accurate result than acting individually. MiR-186-5p acts as target for numerous genes, out of which 15 genes regulate carbohydrate metabolism while others are involved in the HIF-1 signaling pathway; these exist in exosome-free forms. It was found out that miR-186-5p (amongst eight miRNAs) was a very reliable biomarker for CAD and is crucially associated with hypoxia [26]; it thus has the potential to be the most acceptable prognostic biomarker since its level alters post PCI to protect myocardial tissue, therefore offering an accurate prediction of heart health. MiR-186-5p (upregulation) is also known to prove productive during complete vascular obstruction when most of the miRNAs fail to predict the prognosis of patients [27].

## 4. Role of miR-331 and miR-151-3p in CAD

The miR-331 family constitutes a group of three miRNAs (miR-331, miR-331-3p and miR-331-5p). There is sufficient evidence to pillar the pathological as well as physiological role of the miR-331 family in context of vascular heart diseases [28]. The mechanisms how miR-331 executes the regulation of heart diseases will be elucidated. This biomarker would help in well-timed diagnosis of heart diseases and prove a promising contender in the therapeutic plan of action. In most of the studies, peripheral blood samples were worked on, which revealed that miR-331 and miR-151-3p were secreted outside of the myocardium and their levels were notably upregulated during ST-segment-elevation myocardial infarction (STEMI), which mostly is the case during vulnerable plaque rupture [29]. Atherosclerosis (narrowing of artery due to accumulation of plaque) occurs when cytokines, endothelial cells and vascular smooth muscle cells become dysfunctional. It is initiated by high cholesterol and is an inflammatory abnormality in the intima of the artery. This rupturing of atherosclerotic plaque leads to acute cardiovascular diseases. These vulnerable plaques (VPs) have a characteristic large necrosis core and flimsy fibrosis cap, which is the T lymphocytes’ and macrophages’ invasion site. Thus, infiltration of macrophages destabilizes the VPs while autophagy of these macrophages instead stabilizes plaques. These macrophages produce different growth factors, namely, interferon-γ (IFN-γ), matrix metalloproteinase (MMPs), tumor necrosis factor (TNF-α) and cytokines [30]. Thus, the best treatment is to stabilize these VPs [31]. Autophagy (destruction of damaged intracellular parts) helps prevent oxidative injury or cellular distress of plaque cells. Thus, the normal process of autophagy is vital and any disruption in autophagy may increase inflammation [32].

Atherosclerosis can be prevented by blocking phosphoinositide 3-kinase/protein kinase B (PI3K/Akt/mTOR), as depicted in Table 1. This (PI3K/AKT) signaling pathway stabilizes plaques by triggering autophagy. It has been reported that the miR-331 and miR-151 inhibits (PI3K/AKT) the signaling pathway and thus keeps the VP intact [28,33]. Many studies have even unfolded the association of miR-331 and 151-3p with the JAK/STAT pathway. Upregulation of these miRNA upregulates SOCSI (suppressor of cytokine signaling protein, which inhibits both JAK (Janus kinase) and signal transducer but activates STAT transcription of nuclear genes [34].

## 5. Role of miR-29a-3p in CAD

MiR-29a-3p, a member of the miR-29 family, is encoded by a gene located on chromosome 7q32.3. The miR-29 isoforms have emerged as very important molecules that play diverse roles in the gamut of cardiovascular physiology and disease. Deng et al. [38] attributed a protective role to miR-29a-3p in TNFα-induced endothelial dysfunction, which points to miR-29a-3p being a novel target for the prevention and treatment of atherosclerosis. MiR-29a-3p has emerged as a potential therapeutic target in the treatment of atrial fibrillation. Zhao et al. [39] reported that overexpression of miR-29a-3p causes the under expression of the CACNA1C gene (which codes for the α1c-subunit of the L-type calcium channel).

MiR-29a-3p has been shown to be overexpressed in conjunction with other miRNAs in diffuse myocardial fibrosis in patients with hypertrophic cardiomyopathy (HCM), thereby underscoring its role as a biomarker for diffuse myocardial fibrosis in HCM [40]. The members of the miR-29 family, including miR-29a-3p, have been reported to exhibit antifibrotic effects, thereby highlighting its role in post myocardial injuries [41]. MiR-29a-3p has been reported to offer a protective role against reactive oxygen species in patients with acute myocardial infarction. Zhang et al. [42] reported that levels of miR-29a-3p were significantly reduced in acute myocardial infarction patients and cardiac ischemic reperfusion (CIR)-injured mice, and this inhibition of miR-29a-3p induced reactive oxygen species (ROS) production and apoptosis in cardiomyocytes by targeting Bax. Another study found significant upregulation of miR-29a-3p in unstable angina, while no significant changes were observed in myocardial infarction and stable angina [43].

One of the critical contributions of miR-29a-3p is its role in the endothelial function in cardiometabolic disorders. Recently, the analogs of both microR-29a-3p and miR-29b-3p have been reported to improve endothelial function significantly in human type 2 diabetes mellitus arterioles, which imply a potential value for miR-29 mimics as a therapeutic agent for microvascular complications of type 2 diabetes mellitus as well as many other cardiovascular diseases where endothelial dysfunction plays a critical pathophysiological role [44]. Aberrant miR-29 isoform expression is involved in the development of cardiac fibrosis and congestive heart failure whereas a protective function of miR-29a-3p in endothelin-1 (ET-1)-induced cardiomyocyte hypertrophy via inhibiting NFATc4 expression has been reported by Li M et al. [45]. It has been reported that miR-29a-3p along with other miRNAs are associated with the prediction of sudden cardiac death in coronary heart diseases, which could help in the identification of high-risk patients with coronary heart diseases [38].

## 6. Role of miR-574-3p and miR-574-5p in CAD

The gene for miR-574-3p is located on chromosome 4p14. The miR-574-3p has been seen to be overexpressed in CAD patients. The expression of both miR-574-3p and miR-574-5p is upregulated in infarcted heart tissue compared with corresponding remote myocardium in human myocardial infarction, as well as with healthy human hearts [46]. MiR-574-3p has been strongly associated with promoting vascular smooth muscle cell growth in CAD progression [47,48]. In contrast, one previous study has not revealed any association between plasma miR-574-3p and cardiovascular disease [49].

The gene for miR-574-5p is located on chromosome 4p14. The circulating levels of miR-574-5p are upregulated in CAD [49]. MicroR-574-5p expression is increased in myocardial infarcted as compared to healthy human heart tissue [50]. Recently miR-574-5p was significantly upregulated in patients with CAD as compared to healthy controls and also it was demonstrated that the miR-574-5p promoted vascular smooth muscle cell (VSMC) proliferation and inhibited apoptosis [47]. The abnormal proliferation of VSMCs promotes atherosclerotic plaque formation, whereas VSMC apoptosis may promote CAD-related inflammation. It has also been shown that the upregulation of miR-574-5p increased the VSMC growth while its downregulation inhibited the VSMC growth, thus strongly implicating miR-574-5p in CAD pathogenesis [51]. The role of miR-574-5p in the pathogenesis of CAD is proposed to involve the inhibition of ZDHHC14 (a tumor suppressor gene) expression. These reports and other studies suggest that miR-574-5p has potential value as a diagnostic marker and molecular therapeutic target in CAD [46,52].

## 7. Role of miR-1 in CAD

MicroR-1 is predominantly expressed in cardiac tissue and has been found to play an important role in developing cardiovascular diseases [53]. Two genes, namely, miR-1-1 and miR-1-2, are identical in nature and are located in two distinct chromosomal regions in the human genome, 20q13.33 and 18q11.2, which, respectively, encode miR-1 [54]. It has been reported that deletion of either miR-1-1 or miR-1-2 in mice produces similar phenotypes. Accordingly, a miR-1-2-null mouse develops different heart abnormalities, but generally survives as the miR-1-1 gene continuously produces some miR-1 [55]. Similarly; target deletion of miR-1-1 and miR-1-2 in mice produces fatal abnormalities and finally death [56]. The increased expression of miR-1 in the developing heart has been associated with reduced population of ventricular cardiomyocytes [57]. Therefore, a proper level of miR-1 is needed for normal functioning of the heart and any change in its expression level resulted in cardiac diseases. MiR-1 has been associated with different cardiac diseases, such as arrhythmia, myocardial infarction, cardiac hypertrophy and heart failure. Several studies have reported a link between arrhythmia and an abnormal expression of miR-1. A decreased expression level of miR-1 (in the left atria) has been reported in patients suffering from persistent atrial fibrillation [58]. Similarly, severe arrhythmia has been reported with microR-1-2 deletion via Iroquois-class homeodomain protein (Irx5) [59]. A decreased expression of microR-1 has been reported in myocardial infarction (MI) patients and ischemia–reperfusion rats [60].

It has been reported that abnormal expression of miR-1 is associated with cardiomyocyte apoptosis and cardiac hypertrophy development. Sayed et al. [61] showed that the expression of miR-1 was immediately downregulated in cardiac hypertrophy model mice with aortic constriction. They also demonstrated that miR-1 regulates cardiac hypertrophy by negatively regulating the expression of the hypertrophy-associated genes, such as Acta1, Myh7 and Nppa, calmodulin, Mef2a, Ras GTPase-activating protein (RasGAP) and cyclin-dependent kinase 9. miR-1 is also associated with cardiac hypertrophy that is characterized by an increased cell size, heightened organization of the sarcomere and enhanced protein synthesis. It has been reported that miR-1 had significantly higher expression levels in non-ST-segment elevation myocardial infarction or ST-elevated myocardial infarctions than in unstable angina [27]. It has been reported that an increased level of miR-1 is a predictive biomarker for myocardial injury in older individuals [62]; however, this role as a biomarker is still debatable [63].

## 8. Role of miR-208 in CAD

The miR-208 family consists of miR-208a, miR-208b and miR-499 encoded by the myosin genes Myh6, Myh 7 and Myh7b. Tissue distribution of the miR-208 family is different: miR-208a is exclusively expressed in the heart, microR-208b in embryonic heart and miR-499 in skeletal muscles [64,65]. All three miRNAs are intrinsically regulated in a coordinated way. Overexpression of miR-208a has been found to stimulate the expression of miR-208b expression in normal adult mice heart, while silencing of microR-208a expression is associated with the decreased expression of miR-499 and miR-208b in rats [66]. There is strong evidence of a link between the dynamic expression of miR-208a, miR-208b and miR-499 and cardiovascular diseases [67], as depicted in the Table 2.

## 9. Role of miR-223 in CAD

The miR-223 is located on the X chromosome within the arm q12 locus, and several transcription factor, viz., transcription factor PU.1, nuclear factor I-A (NFI-A) and CCAAT-enhancer-binding proteins- (C/EBP-) *α* and *β*, regulate its expression [68]. MiR-223 is found in the form of most abundant miRs in platelet and HDL and it is found as a form of an Ago2–miR-223 complex in platelet microparticles (MPs). Human platelets activated by thrombin preferentially release Ago2–*miR-223* complexes in MPs, which regulate the expression of endothelial mRNA FBXW7 and EFNA1 after being released from the MPs, showing that activated platelets deliver mRNA regulatory Ago2–miR complexes to other cells and regulate the expression of the endogenous genes in the recipient cell, such as endothelial cells (ECs) [69]. Vickers et al. found miRNAs in human high-density lipoprotein (HDL) and revealed a new mechanism of intracellular signaling through transported miRNAs to other cells [70]; also, the subjects with familial hypercholesterolemia of HDL-miRNA profiling were significantly different from those of the controls, which suggest that the quality as well as quantity of HDL has a most important role. Furthermore, one of the studies reported the anti-inflammatory function of HDL regulated by miRNAs, in which miR-223 in the HDL was transported to the ECs and decreased the expression of ICAM-1 in the ECs [71]. On the other hand, the same study revealed that HDL-miRNA from atherosclerotic patients induced differential gene expression in hepatocytes [72].

## 10. Role of miR-155 in CAD

The gene for miR-155 is located on chromosome 21q21.3 and its deregulation has been shown to be related to different forms of cancer, cardiovascular diseases and viral infections [73]. Zi-Liang Ye et al. studied the association between the expression of miR-155 in peripheral blood CD4^+^ T lymphocytes and the level of serum interferon-γ (IFN-γ) concentration and the severity of CAD and concluded that the level of miR-155 and the level of IFN-γ are closely correlated with the severity of CAD [74]. 

A recent study showed that both miR-155 and miR-221/222, which are highly expressed in endothelial cells (ECs), control Ets-1, induced by Ang II negatively. In addition, the overexpression of miR-155 or miR-221/222 in HUVECs downregulates the vascular cell adhesion molecule 1 (*VCAM*-*1*), monocyte chemoattractant protein-1 (*MCP*-*1*) and Fms-related receptor tyrosine kinase 1 (FLT-1) mRNA expressions caused by Ang II [75,76]. It has been reported that that the miR-155 regulates angiotensin II type 1 receptor expression in human umbilical vein endothelial cells [77]. Sun et al. indicated that miR-155 induced the adverse functions at ECs and atherosclerosis [78]. MiR-155 mediates endothelial inflammation and decreases the expression of the nuclear factor-kappa B (NF-B) p65 and adhesion molecules such as ICAM-1 or VCAM-1 in TNF-treated ECs [79]. It was suggested that in ECs miR-155 has protective functions. In patients with CAD, miR-155 circulation is significantly decreased as compared with healthy controls [80]. It has been found that eNOS is a direct target of miR-155 [78,81]. The inflammatory cytokines, including tumor necrosis factor-α, increased miR-155 expression [82]. MiR-155 inhibition opposes TNF-α-induced downregulation of endothelial nitric oxide synthase, and the impairment of endothelium-dependent vasorelaxation [78]. MiR-155 has been described as a powerful marker for detecting CAD [83].

## 11. Role of miR-423 in CAD

MiR-423-5p is localized in chromosome 17q11.2 in the intron of the nuclear speckle splicing regulatory protein 1 gene [84]. It has been reported that there is a difference in the microR-423-5p trans-coronary gradients between patients with stable systolic heart failure and healthy controls, and it is suggested that miR-423-5p originates from heart tissues [85,86]. It has been shown that the microR-423-5p serum levels were significantly higher in patients with unstable angina pectoris than patients with stable angina pectoris and aortic stenosis [87,88]. It is suggested that the serum levels of miR-423-5p may reflect the stage of myocardial ischemia. Moreover, miR-423-5p has been suggested as a potential biomarker in cardiac failure and CAD [89,90]. It has been proposed that the miR-423-5p plasma levels are positively associated with the peripheral N-terminal (NT) pro–B-type natriuretic peptide (pro-BNP) [91]. The N-terminal (NT) pro–B-type natriuretic peptide is a marker of hemodynamic stress; its serum levels are increased in subjects with increased left ventricular mass, CAD and peripheral artery disease [92]. Furthermore, it has been shown that miR-423-5p predicts 3 months death in cardiogenic shock and that elevated miR-423-5p is a marker of hypoperfusion [93]. Nabiałek et al. have also suggested circulating miR-423-5p as a potential early marker of myocardial infarction; they reported that the miR-423-5p plasma level is significantly increased in early AMI; then its level approaches normalization within the next 360 min [94]. Rizzacasa et al. have examined the expression profile of more than 80 microRNAs in the blood of CAD cases using real-time PCR and have shown that miR-423-5p exhibits different expression in plasma and peripheral blood mononuclear cell of subjects with stable and acute MI [95]. This pilot study stated the potential role of miR-423-5p as a new epigenetic biomarker in diagnosis and prognosis of CAD and AMI. However, a similar study reported that the serum level of miR-423 is reduced within the first day of the AMI and increased for 180 days after the AMI [96]. It has recently been indicated that miR-423-5p has been shown to directly regulate the O-GlcNAc transferase (OGT) gene in cardiomyocytes in a study conducted in mice [97]. Barkovskaya et al. reported that the GlcNAc transferase (OGT) catalyzes the addition of sugar β-N-acetylglucosamine (O-GlcNAc) sugar onto Ser and threonine residues of proteins substrate; the (O-GlcNAc) donor is the UDP-GlcNAc and also the OGT is essential for cell division and embryogenesis [98]. The expression of miR-423-5p inhibits of GlcNAc transferase expression and its downstream target, AMPK phosphorylation. Then, the apoptosis-promoting proteins, proteins p53 and caspase-3 will be upregulated, leading to an increased apoptotic rate of cardiomyocytes. Therefore, miR-423-5p transfection results in cardiomyocyte apoptosis [99]. It has been predicted that miR-423-5p may regulate the transcription factors involved in important physiological processes, such as proliferation and differentiation [95].

## 12. Role of miR-133b in CAD

In humans, three miR-133 genes, miR-133a-1, miR-133a-2 and miR-133b, are located on chromosomes 18, 20 and 6 respectively. miR-133 is highly expressed in myocardium and skeletal muscle, and is involved in the development, differentiation, survival and electrical conduction of cardiomyocytes [100]; it is also implicated in cardiac hypertrophy, cardiac fibrosis, cardiac hypertrophy and heart arrhythmia, and it has been reported that miR-133 targets genes involved in cardiac hypertrophy, such as RhoA, Cdc42, Nelf-A/WHSC2, MAPK, TGFβ/Smad and PI3K/Akt [101].

This study concluded that miR-133 is a regulator of cardiac hypertrophy and proposed a therapeutic application. miR-133a and b have been reported to be upregulated in patients with CAD [102]. It has been reported that patients with left ventricle diastolic dysfunction have reduced miR-133 levels compared to healthy controls. Moreover, Kuwabara et al. [103] reported that the miR-133a level can be used a biomarker for cardiomyocytes necrosis; they also reported that miR-133a is elevated in cases with AMI, unstable angina pectoris and Takotsubo cardiomyopathy, and that the miR-133a level increased from damaged myocardium. An increased circulating miR-133a level in cases with ST-elevation myocardial infarction is correlated with decreased myocardial salvage, more infarction size, obstruction of the microvasculature and more distinctive reperfusion damage. Moreover, miR-133a and miR-133b have been described among the circulating miRNAs in various cardiovascular diseases [104]. Kumar et al. [105] compared the plasma levels of miR-133b in 15 pre-atherosclerotic normal coronary artery cases, 78 CAD subjects and 54 healthy controls, and reported that miR-133b is highly enriched in the myocardium. There is a negative correlation between the miR-133b plasma levels and the risk of CAD and therefore concluded that miR-133b is a potential biomarker to predict different stages of the CAD from the stage of pre-atherosclerosis to the advanced atherosclerotic condition.

## 13. Role of miR-30 in CAD

The miR-30 (miR-30) family is one of main members of the miRNAs, consisting of five members and six mature miRNA molecules, which are miR-30a, miR-30b, miR-30c-1, miR-30c-2, miR-30d and miR-30e. These are encoded by six genes located at three different chromosomal regions: 1p34.2 (miR-30c and miR-30e), 6q13 (miR-30a) and 8q24.22 (miR-30b and miR-30d). These six mature miRNAs possess a similar sequence near the 5′ end but differ by having a compensatory sequence near the 3′ end, which allow the miR-30 family members to perform different biological functions. The miR-30 members act as key regulators of complex biological processes in multiple CVDs, including ischemic heart disease, heart failure, hypertension and arrhythmias [105,106]. Furthermore, due to their presence in the circulation after the cardiovascular pathologies, they have been explored as novel biomarkers, particularly in the milieu of AMI and HF.

The miR-30 family is predominantly expressed in the heart, and its expression levels are decreased after cardiomyocyte hypertrophy and MI; they also play a role in ventricular remodeling (VR) [107,108]. The potential role of miR-30 in various physiological and pathological states has been observed and the increasing evidence shows that the miR-30 family also plays a crucial role in regulating autophagy, apoptosis, oxidative stress and inflammation. Interestingly, miR-30 released by cardiac fibroblasts acts as a paracrine mediator of cardiomyocyte hypertrophy; this makes the miR-30 family an attractive diagnostic and prognostic biomarker in the cardiovascular field. The miR-30 family can be easily assessed, with a robust stability in the circulating blood or plasma and with an excellent sensitivity [109,110]. The downregulation of the miR-30 family could be related to the rarefaction process and hypertension, making it a prognostic maker. Many studies have investigated the role of miR-30 in the pathogenesis of CVDs, making it one of the attractive diagnostic and prognostic biomarkers and giving a platform for the development of novel therapeutic strategies for CVDs [106,111].

## 14. Role of microR-147b in CAD

MiR-147b (miR-147b or hsa-miR-147b) is one of the key miRNAs found in humans and its gene is located on chromosome number 15. It is involved in posttranscriptional regulation of gene expression by affecting both the stability and translation of the target miRNAs. Studies demonstrated the participation of miR-147 in a negative feedback loop that is able to inhibit the proinflammatory response of macrophages to multiple TLR ligands. When dysregulated, its role has been seen in cancers, suggesting its oncogenic role; its role has also been confirmed in CVDs [112]. Chatterjee et al. reported a novel function of miR-147b in protecting barrier function in human vascular endothelial cells, which affects CVDs, and expression of microR-147 in CAD patients was reported less compared to the controls [113]. Yao-Meng et al. [109] reported lower expression miR-147 in patients with dilated cardiomyopathy (DCM) compared with control subjects, suggesting its role as a potential diagnostic biomarker [114]. Mingxia Gu et al. have shown that miR-147b inhibits viability and promotes cell apoptosis, which has a role in the pathogenesis of heart failure [115]. This makes miR-147b one of the important diagnostic biomarkers, as depicted in Table 3.

## 15. Role of miR-638 in CAD

The miR-638 gene is located on chromosome 19 and it plays is an important miRNA, playing key roles in physiological processes in humans, such as development and transcriptional modification. The dysregulation of miR-638 has been seen in many pathologies, including cancer, lupus nephritis (LN) and CVDs. miR-638 participates in the pathophysiology of CVDs, such as stroke, and therefore is a potential biomarker of CVDs [116].

Luque et al. [112] showed lower serum miR-638 in symptomatic patients with carotid stenosis undergoing a carotid endarterectomy [117]. Serum miR-638 determination may also be useful for long-term management, monitoring and atherothrombotic plaque identification. So, the detection of miR-638 in serum may constitute a potential noninvasive biomarker associated with ischemic stroke, particularly in high cardiovascular risk individuals. Pan Li et al. showed that miR-638 is most significantly downregulated in human vascular smooth muscle cell (VSMC) proliferation [118]. Wong et al. showed miR-638 distinguished heart failure with reduced vs. preserved left ventricular ejection fraction and offers better diagnosis than N-terminal pro-brain natriuretic peptide [119]. Jiao et al. indicated that miR-638 is upregulated in human calcific aortic valves (hAVICs) compared with non-calcific valves and was significantly upregulated during hAVICs osteogenic differentiation [120]. This constitutes a promising noninvasive diagnostic as well as prognostic biomarker in cardiovascular pathologies risk.

## 16. Conclusions

In this review, we address the present state of understanding on the biogenesis, regulation and pathophysiological roles of miRNAs in cardiovascular diseases, and the potential future perspectives on their use as biomarkers and therapeutic agents. They are potent biomarkers as they can be easily detected in body fluids (blood, urine, etc.) due to their remarkable stability; they are also present in apoptotic bodies and exosomes. Existing studies suggest the role of microRs as valuable biomarkers. The miRNAs have many characteristics of an ideal biomarker, most notably their inherent stability and resilience, which accordingly make them promising candidates for the development of diagnostic tools and therapies for cardiovascular disease. A single biomarker may be insufficient to diagnose CAD or AMI; thus, a combination of different miRNAs may prove fruitful in the diagnosis and stratification of CVDs. Early diagnosis of CVD remains a challenge for clinicians; it is an important goal to reduce treatment-associated morbidity and mortality, and reach maximal long-term survival. Therefore, there is a pressing need to develop cost-effective, noninvasive and accurate screening of alternative diagnostic techniques for cardiovascular disease. Recent blood-based miRNA profiling studies, reporting their presence in serum and plasma, have generated the concept that circulating miRNAs hold much potential as novel noninvasive biomarkers for many diseases, including cardiovascular disease.

## Figures and Tables

**Table 1:** List of miR-19b-3p, 134-5p, 186-5p, 331 and 151-3p and their target genes and regulation factors, effects or pathways.

microRNA	Target	Regulation Factors/Effects/Pathways	References
miR-19b-3p	PGC-1α, ABCA1,TF (procoagulant protein), Arigin I, MuRF I (muscle RING finger protein 1),TGF*β*RII	Accumulation of foam cells and macrophages, calcineurin/NFAT PKC/TGF*β*signaling	[35]
miR-134-5p	PI3K/Akt/mTOR, CREB A12B (HSPA12B)	Interferon-γ (IFN-γ), matrix metalloproteinase (MMPs), tumor necrosis factor (TNF-α) and cytokines	[36]
miR-186-5p	PI3K/Akt/mTOR	Interferon-γ (IFN-γ), matrix metalloproteinase (MMPs), tumor necrosis factor (TNF-α) and cytokines	[37]
miR-331	SOCS1	JAK and signal transducers gets inhibited STAT gets activated	[24]
miR-151-3p	SOCS1	JAK and signal transducers gets inhibited STAT gets activated	[25]

Legend: PGC-1alpha—peroxisome proliferator-activated receptor gamma coactivator 1-alpha; ABCA1—ATP binding cassette subfamily A member 1; TGF*β*RII—transforming growth factor beta receptor II; NFAT—nuclear factor of activated T-cells; P13K/Akt/mTOR—phosphatidylinositol-3-kinase a serine/threonine protein kinase mammalian target of rapamycin; CREB—cAMP response element-binding protein; PKC—protein kinase C; SOCS1—suppressor of cytokine signaling 1; HSPA12B—heat shock protein family A (Hsp70) member 12B.

**Table 2:** Summary of the miR-208 family in cardiovascular disease.

miRNAs	Species	Diseases	Phenotype
miR-208a	Human	AM	Over-expressed
	Rat	Myocardial injury	Over-expressed
	Human	AMI and AP	Over-expressed
	Human	HF	Over-expressed
	Rat	High-salt diet	Downregulated
	Human	DCM	Over-expressed
	H9C2	Mechanical stretch	Downregulated
	Rat	AV shunt	Over-expressed
	Human	AMI	Over-expressed
miR-208b	Human	AMI	Over-expressed
	Human	AMI	Over-expressed
	Human	Acute STEMI	Over-expressed
	Human	Acute STEMI and NSTEMI	Over-expressed
	Rat	High-salt diet	Over-expressed
	Mouse	TAB	Over-expressed
miR-499	Human	AMI and acute HF	Over-expressed
	Human	AMI	Over-expressed
	Human	Acute STEMI and NSTEMI	Over-expressed
	Human	AMI and AP	Over-expressed
	Human	AF	Over-expressed
	Human	HF	Over-expressed
	Human	Acute NSTEMI	Over-expressed

AMI: acute myocardial infarction; VF/VT: ventricular fibrillation/ventricular tachycardia; AV block: atrioventricular block; AF: atrial fibrillation; AP: angina pectoris; HF: heart failure; DCM: dilated cardiomyopathy; TAB: thoracic aortic banding; AV shunt: aorta-caval shunt; STEMI: ST segment elevated myocardial infarction; NSTEMI: non-ST-segment elevated myocardial infarction.

**Table 3:** Diagnostic and prognostic role of various the miRNAs (miR-30, miR-147b and miR-638) associated with cardiovascular disorders (CVDs).

miRNA ID	Change in Expression	Purpose	Pathology
miR-30a	Increased	Diagnostic	MI
miR-30b	Decreased	Diagnostic	AHF
miR-30c	Decreased	Diagnostic	AMI
	Decreased	Diagnostic	Fibrosis
miR-30d	Decreased	Diagnostic and prognostic	CHF
miR147	Decreased	Diagnostic	AP
miR-638	Decreased	Diagnostic and prognostic	CS
	Increased	Diagnostic	hAVICs

## Abbreviations

CAD	Coronary artery disease
CVD	Cardiovascular diseases
MCI	Myocardial infarction
AMI	Acute myocardial infarction
CVDs	Cardiovascular disorders
HDL	High-density lipoprotein
ECs	Endothelial cells
NFI-A	Nuclear factor I-A
MCP-1	Monocyte chemoattractant protein-1
FLT-1	Fms-related receptor tyrosine kinase 1
HUVECs	Human umbilical vein endothelial cells
VCAM-1	Vascular cell adhesion molecule 1
OGT	O-GlcNAc transferase
VR	Ventricular remodeling
DCM-	Dilated cardiomyopathy
hAVICs	Human calcific aortic valves
VSMC	Vascular smooth muscle cell
ECs	Endothelial cells
RasGAP	Ras GTPase-activating protein
HCM	Hypertrophic cardiomyopathy
ROS	Reactive oxygen species
MMPs	Matrix metalloproteinase
IFN-γ	Interferon-γ
TNF-α	Tumor necrosis factor
VP	Vulnerable plaques
STEMI	ST-segment-elevation myocardial infarction
LOX	Lysyl oxidase
DGCR8	DiGeorge syndrome critical region 8
ICAM-1	Intercellular adhesion molecule 1
eNOS	Endothelial nitric oxide synthase 3
MAPK	Mitogen-activated protein kinase
C/EBP	CCAAT-enhancer-binding proteins α and β
CHF-	Congestive heart failure
